# Report of the Prime Minister's Committee

**Published:** 1918-05-25

**Authors:** 


### THE POSITION OF SCIENCE IN EDUCATION.

### REPORT OF THE PRIME MINISTER'S COMMITTEE.

### III.—Abolish Present Waste of Intellect and Provide Better System.

Among the subjects into which the Committee inquires is the future supply of medical students, and what it says on this matter is worthy of serious attention. The Committee quotes the opinion of Sir Donald MacAlister, the Chairman of the General Medical Council, that though there will undoubtedly be a shortage in the number of practitioners added to the Register in 1918 and 1919, he did not think that this shortage would be more than temporary. The Committee finds it, however, extremely difficult to discover the present sources of this future supply in England, though the promise is much greater in Scotland, owing to the custom therein obtaining for boys of ability who have passed with credit through a secondary school to satisfy their ambition by entering the medical profession. In England, of 230 students who entered the medical profession in 1913 through the portal of the Universities, only forty-seven came from grant-earning schools. In other words, the great majority of future medical practitioners were recruited from families of the well-to-do.

The chief reason is to be found, no doubt, in the *res angusta domi.* A medical education is very long, and very expensive; and however able a boy may be, his parents, if without capital, prefer to train him for some occupation in which he will be securely provided for on graduation. Again, a boy educated in a grant-earning school, though he might make an excellent practitioner of medicine, has often no one to guide him through the difficulties that surround entry into the medical profession. A third reason, which is not given by the Committee, but which we believe to operate in placing medicine at a disadvantage in comparison with other professions, is the absence of any of those great prizes that may captivate the imagination and inspire the ambition of the young. The entrant into the Church may aspire to a bishopric, or even to the position of second person in the realm, immediately after the Sovereign himself. The entrant into the Law has before his eyes the possibility of a judgeship, or even of the dazzling position of a Lord Chancellor. The cadet on entering Woolwich or Sandhurst packs a Field-marshal's baton in his kit. The boy entering Osborne sees at the end of a long vista the position of Admiral of the Fleet. But the budding medical practitioner can look no higher than the Presidency of a professional College, or perhaps a seat upon a borough bench of magistrates. The positions are honourable, no doubt, but they are scarcely such as to fire the ambition of the youthful aspirant to fame.

Is the entry of women into the profession of medicine likely to compensate for the dearth of male candidates? Since the war broke out, there has been a great influx of women into the medical schools. It does not appear to the Committee likely that this great rush will continue in time of peace, but the Committee gives no reason for this opinion, and it admits that the multiplication of posts for women practitioners and the probable further increase of such opportunities will attract a much larger number of women into the profession than before the war.

The vast majority of women students of medicine come from proprietary schools, such as those of the Girls' Public Day School Trust, and enter the profession by taking a matriculation examination at one of the Universities, or one of the equivalent Oxford and Cambridge examinations; but very few of the girls who have won a county council scholarship finally pass into medicine. They are repelled chiefly by the cost of a medical education, which, apart altogether from living expenses, will certainly reach £250. There are many women, says the Committee, who would make excellent members of the medical profession, though they cannot face the expense of a medical training. To meet such cases the Committee proposes that more scholarships, tenable throughout the course of medical education, should be available, and they add that such scholarships are needed both for men and for women. We very heartily agree. It is well known that the most brilliant practitioner of medicine of the last generation owed his entrance into the medical profession and his medical education to private eleemosynary assistance; but we do not know how many William Gulls are prevented by narrowness of means from entering and adorning the medical profession.

In this connection an excellent suggestion is made by the Committee. The award of scholarships is not the only method by which assistance can be given to students. In the later stages of professional education, when a student is approaching the time when he will be able to earn money, much help can be given without great expenditure by the establishment of loan funds. This has proved very valuable at some of the women's colleges, and at the Medical School for Women.

The object, or one great object, of a good system of education, such as we have not, but such as we should have if the recommendations of the Prime Minister's Committee were put in practice, is to prevent the waste of intellect that is permitted and encouraged by the present system of education. The waste is twofold. The ability that is discovered is sterilised, repressed, or misdirected by a bad educational system; and much ability that might be utilised in more highly intellectual occupations remains undiscovered, and is wasted upon manual labour, or upon clerical or other occupations that make little call upon the intelligence. How great this waste is, and how easily it might be prevented,

☛ Previous articles appeared on May 11 and 18, p. 139.

**POSITION OF SCIENCE IN EDUCATION—(cont.).**

is shown by the results of the Admiralty scheme of training dockyard apprentices. In the dockyard schools, recruited from elementary or municipal secondary schools, and to which boys are admitted by open competition, and while serving as apprentices, the standard is maintained at a high level by annual rejections on the results of examinations. From these schools have come many holders of Royal and Whitworth scholarships, many distinguished naval architects and engineers, and a large proportion of the Directors of Naval Construction. The lesson is most striking and significant, and gives us some notion of the immense store of unutilised ability that exists among us and is wasted for want of proper opportunities.

It is of the first importance, says the Committee, that ability should not be wasted, and if it is not to be wasted, measures must be taken to ensure (1) that no pupil capable of profiting by a full secondary education should miss the opportunity of receiving it, and (2) that the leakage from the schools should be, as far as possible, stopped. By leakage from the schools the Committee means the premature leaving of school before school education is completed.

The guiding principle of the Report is, it will be seen, not only to provide for the introduction into the curriculum of every school of natural science as an integral, necessary, and important part of education; not only to make provision that instruction in natural science and training in scientific methods should be made universal, and carried out throughout the whole of the formal education of every man and woman, from its commencement in the elementary or preparatory school to its termination by the attainment of a degree at a university; but also to search out and discover intellectual ability wherever it exists, and when it is discovered to give to it the best possible education, to see that that education is continued to the end, and is not prematurely cut short by want of means, by the indifference, greed, or poverty of parents, or by any other removable cause.

Nor do the investigations of the Committee or their recommendations end at the attainment of a university degree by the student. They pursue him into his post-graduate career, and lay down the conditions that ought to be observed in order to foster original research. To this end the provision of research scholarships is advised. Indeed, throughout the Report an abundance of scholarships at every stage of education is recommended, and the recommendations seem eminently wise from the point of view of the welfare of the nation, which is the governing motive of the Report. Before the war, such recommendations would have been made to deaf ears, but the war has not only brought into striking prominence the defects in our educational system and the need for utilising all the ability to be found in the nation, but it has also familiarised us with expenditure upon a colossal scale; and expenditure that before 1914 would have been scouted as extravagant and impossible has now a chance of being seriously considered, even when it is to be upon education. After all, the nation spends upon its universities less than half the sum that it spends upon its prisons.

The whole scheme is calculated and intended to encourage, and indeed to make compulsory, the cultivation of science in education; but it is far more than this. The members of the Committee are, as they themselves declare, keenly alive to the necessity for developing the literary training of the student, and again and again they insist on the importance of securing for him an ability to express himself in his own tongue. The whole scheme is directed to secure for every English boy and girl the education that is most suitable, and the one that can best develop to the utmost his or her innate capacity; and it seems to us eminently calculated to achieve these purposes.

